# The Turkish version of the SPPIC validated among informal caregivers with a Turkish immigrant background

**DOI:** 10.1186/s12877-021-02161-6

**Published:** 2021-04-29

**Authors:** Nienke van Wezel, Iris van der Heide, Walter L. J. M. Devillé, Gozde Duran, Rianne Hoopman, Marco M. Blom, Anne Margriet Pot, Peter Spreeuwenberg, Anneke L. Francke

**Affiliations:** 1 Alzheimer Nederland, Amersfoort, Netherlands; 2 Netherlands Institute for Health Services Research (Nivel), Utrecht, Netherlands; 3 Amsterdam Institute for Social Science Research, University of Amsterdam, Amsterdam, The Netherlands; 4 Julius Center for Health Sciences and Primary Care, University Medical Center Utrecht, Utrecht, Netherlands; 5 Department of Epidemiology and Biostatistics, Amsterdam Public Health Research Institute VU University Medical Center, Amsterdam, The Netherlands; 6 School of Psychology, University of Queensland, Brisbane, Australia; 7 World Health Organisation, Geneva, Switzerland; 8 Amsterdam UMC, Vrije Universiteit Amsterdam, Department of Public and Occupational Health, Amsterdam Public Health Research Institute, Amsterdam, The Netherlands

**Keywords:** Dementia, Family caregivers, Self-perceived pressure from informal care, Questionnaire validation, Migrants

## Abstract

**Background:**

This study assesses the internal consistency and known group validity of the Turkish version of the SPPIC, a measurement instrument to assess the self perceived pressure from informal care in family caregivers of people with dementia that was originally in Dutch.

**Methods:**

The feasibility, comprehensibility and appropriateness of the Turkish SPPIC were assessed during a pilot test. Internal consistency was examined based on data from 117 family caregivers with a Turkish immigrant background by calculating Cronbach’s alpha and by conducting a single-factor Confirmatory Factor Analysis (CFA). Known group validity was determined to obtain an understanding of the validity of the translated instrument, testing differences in the self-perceived pressure from informal care, depending on frequency of caregiving, living with a person with dementia and level of education.

**Results:**

The pilot test showed that the translated SPPIC was considered to be feasible, comprehensible and appropriate. The internal consistency appeared to be strong (Cronbach’s alpha: 0.94). The CFA indicated that the factor ‘Self-perceived Pressure from Informal Care’ explained varying levels of variance in the items of the SPPIC (ranging from .52 to .87). Family caregivers who provided care at least once a week and who shared a home with a person with dementia perceived a greater pressure from informal care (*p* = 0.007, *p* = 0.001).

**Conclusions:**

The Turkish translation of the SPPIC can be used in future research and practice to obtain insight into self-perceived pressure from informal care of family caregivers with Turkish immigrant backgrounds. At the same time it is recommended to conduct more research on how the measurement of self-perceived pressure from informal care in this group can be further improved.

**Supplementary Information:**

The online version contains supplementary material available at 10.1186/s12877-021-02161-6.

## Background

Studies show that family caregivers often perceive caregiving as stressful or burdensome, especially those who take care of a person with dementia [1–3]. Compared to other family caregivers, family caregivers taking care of a person with dementia are more often overburdened [4–7]. Many studies show that the stress and pressure as a consequence of caring for a person with dementia can lead to poor health outcomes in family caregivers, including depression [8, 9]. In order to offer timely support and thereby prevent overburdening in family caregivers, it is important to have insight into their self-perceived care pressure. The model of carer stress and burden, as published by Sörensen and colleagues [10], combines several theoretical models of carer burden and stress, and is a commonly used theoretical framework for guiding caregiving research [10]. It entails well-documented primary and secondary stressors as well as background and contextual factors that relate to care burdens in family caregivers of people with dementia. A primary stressor in this model is the severity of the disease. As dementia progresses, problem behaviour as well as cognitive and functional impairment tend to worsen, increasing the pressure on family caregivers. Furthermore, the care situation, including the hours of care and the duration of care, is also one of the primary stressors. Spousal caregivers, sharing a home with a person with dementia, often provide long-term care on a day-to-day basis and are therefore more likely to experience a high self-perceived pressure from informal care than caregivers who live separately from the person with dementia [11]. Background and contextual factors that account for a higher self-perceived pressure from informal care in family caregivers, according to the model of carer stress and burden, include having a lower socioeconomic status (and therefore fewer resources) [10, 12–14], being older, being a female caregiver and having a specific ethnic or cultural background compared to other ethnic groups [15].

Assessing the self- perceived pressure from informal care can help recognize those family caregivers who are especially in need of support. Various measurement instruments have been developed to assess self- perceived pressure form informal care among family caregivers [16–18]. A validated and frequently used Dutch questionnaire for measuring the self- perceived pressure form informal care of family caregivers of people with dementia is the SPPIC (Self-perceived Pressure from Informal Care) [17]. The SPPIC was originally developed and validated in Dutch in 1995. The SPPIC measures the demands of the care situation as perceived by the family caregiver and in relation to the caregiver’s needs, such as time for other activities [19]. However, this version of the SPPIC is only available in Dutch. A Turkish version of the SPPIC is highly desirable as 12.7% of the Dutch population has non-Western immigrant backgrounds [19] and people with a Turkish background are the largest group within that category (https://www.cbs.nl/nl-nl/achtergrond/2016/47/bevolking-naar-migratieachtergrond). The first generation of immigrants with Turkish background have now reached the age at which dementia becomes increasingly prevalent. We assume that the self- perceived pressure form informal care in family caregivers with a Turkish background might be relatively high because (a) the care for a family member with dementia is preferred to be provided within the family circle, (b) beliefs regarding severe memory loss and ageing might make people refrain from seeking professional support, and (c) because the options for professional care and support are often not known [20].

For these reasons, we developed a supportive peer-group-based educational intervention to enhance knowledge about the disease dementia and about care and support options for family caregivers with an immigrant background [21]. We aimed to study the effects of this intervention on self- perceived pressure form informal care in family caregivers with a Turkish background. The translation and validation of the SPPIC in Turkish were part of this larger study, which included a pilot phase before the main study in order to test the feasibility, comprehensibility and appropriateness of the translated measurement instruments, including the SPPIC. The aim of the current study is to examine the internal consistency and the known group validity of the Turkish version of the SPPIC.

## Method

### Translation of SPPIC

The SPPIC consists of nine statements about the care provided by the family caregiver (see Appendix 1). Each statement can be answered with ‘No!’, ‘No’, ‘More or less’, ‘Yes’ or ‘Yes!’ To give an example, one of the statements is “I must always be available for my …” To translate the Dutch SPPIC we used the principles of forward and back-translation [22]. The nine statements were first translated from Dutch into Turkish by a professional Turkish native-speaking translator. After that, the Turkish version of the SPPIC was translated back into Dutch. The original Dutch version was then compared against the back-translated Turkish version by one of the research group members who is a native Turkish speaker. The research group members discussed some minor differences in the nuances of the translations and the wording was amended accordingly.

### Pilot test: feasibility, comprehensibility and appropriateness

To determine the feasibility, comprehensibility and appropriateness of the translated items of the SPPIC, a pilot test was conducted among 30 Turkish first or second-generation family caregivers aged 25–72 whose level of education ranged from none to a university degree. The participants in the pilot test were recruited in community centres in a large city in the south of the Netherlands (Tilburg). This region was not part of the overall study. Participants were offered the choice of filling in the Dutch or the Turkish version of the questionnaire. All thirty participants completed the Turkish version of the SPPIC. The research staff then made a verbal inventory of whether the participants correctly understood the items (comprehensibility), whether the items were difficult to answer (feasibility) and whether the items were seen as relevant for assessing the self-perceived pressure from informal care (appropriateness). This inventory showed that no adaptations of the items of the SPPIC were needed. The Dutch version as well as the English translation are included in Appendix 1.

### Main study: internal consistency and validity

#### Participants

The internal consistency and validity of the Turkish version of the SPPIC were assessed in the context of an intervention study that was set up to evaluate the effects of a peer-group-based educational intervention for family caregivers with an immigrant background. The participants for this intervention study were recruited in two provinces of the Netherlands, in which no peer-group-based educational intervention was offered before and where relatively many people live with a Turkish immigrant background (https://www.cbs.nl/nl-nl/achtergrond/2016/47/bevolking-naar-migratieachtergrond). Participants were recruited through key figures in the communities (such as community workers, imams, ethnic minority senior citizen advisers, ethnic minority care organizations and regional branches of the Dutch Alzheimer Association). These key figures asked people in their network who had a relative with dementia or severe forgetfulness whether they would be willing to take part in the peer-group based educational intervention. The key figures gave a verbal explanation and provided written information about the intervention and the associated study and inclusion criteria. If family caregivers wanted to take part, the key figures then passed on their contact details to the research coordinator. The coordinator assessed (by means of a short oral intake interview with each participant) whether the family caregivers who had expressed an interest met the inclusion criteria. The following inclusion criteria were applied to select participants with a Turkish background:

- must have a relative or loved one with dementia or – if there has not yet been a formal diagnosis – with severe forgetfulness;
- must have been born in Turkey or have at least one parent born in that country;
- must live in the Netherlands;
- must be able to complete a written questionnaire independently or to complete the questionnaire with the aid of a trained research assistant;
- must not be suffering from severe forgetfulness or dementia themselves.

#### Procedure

Only data from the baseline measurements, i.e. the measurements before the start of the peer-group based educational intervention, among participants who filled in the Turkish version of the SPPIC were used for the psychometric analyses described in this article. Participants who were literate were asked to fill in the questionnaire themselves. Participants could choose whether they wanted to complete the questionnaire in Dutch or in Turkish. Research assistants with a Turkish background were available to help participants who were not literate. For those participants, the research assistants read out the questions and scored the statements according to the answers given by the participant. Prior to participation, the research coordinator gave the participants an information letter about the study together with a consent form. These were available in Turkish and in Dutch. All participants gave their informed consent in writing. In the case of illiterate participants, a research assistant who spoke their mother tongue read out the information letter and consent form.

#### Ethical approval

Under Dutch law, approval from a medical ethics committee or social/societal ethical committee was not required for this study as the participants were mentally competent, they were not subject to the imposition of a certain kind of behaviour and they were not subjected to burdensome interventions or measurements (https://english.ccmo.nl/investigators/legal-framework-for-medical-scientific-research/your-research-is-it-subject-to-the-wmo-or-not).

#### Assessments

The following sociodemographic variables were assessed by a questionnaire: sex, age, highest completed level of education (none or primary school, secondary school, secondary vocational education, higher professional education or university, or other) and country of birth. In addition to that, characteristics related to familiarity with dementia were assessed: whether dementia is present in the family, whether the respondent lived together with a person with dementia, whether the respondent provided care (personal care, domestic help, practical help, providing a listening ear, watching over, nursing care and companionship), how often the respondent provided help (daily, 3–6 times a week, up to twice a week, less than once a week, less than once a month). The language proficiency in both the mother tongue and Dutch were also assessed for reading, writing, understanding and speaking (none, little or good). Answers to the nine items of the SPPIC were recoded to a numeric score, ranging from 1 to 5 per item. Sum scores were subsequently calculated ranging from 9 (the lowest self-perceived pressure of informal care) to 45 (the highest self-perceived pressure of informal care).

#### Statistical analyses

Descriptive statistics were used to describe the scores on the items of the SPPIC. The internal consistency of SPPIC was examined by calculating correlation coefficients between the items of the SPPIC and the Cronbach’s α across the items (with an α of ≥0.7 indicating adequate internal consistency) [22]. Subsequently, confirmatory factor analysis (CFA) was conducted using structural equation modelling to determine whether all nine items of the SPPIC reflected a single homogeneous dimension of ‘self- perceived pressure form informal care’, as suggested in the original validation study of the SPPIC [17]. The extent was therefore tested to which the nine items loaded on a single factor and to what extent this single factor model fitted the data. The goodness of fit was used to evaluate how well the proposed single-factor model fitted the data. χ^2^ is a statistic for evaluating the overall model fit [22, 23]. A non-significant χ^2^ value suggests that the hypothesized model fits the data. Furthermore, Comparative Fit Index (CFI), and Tucker-Lewis index (TLI) were used to assess the model fit. Values of < 0.90 indicate no fit; values between 0.90 and 0.95 indicate acceptable fit; values of > 0.95 suggest an excellent fit [22, 23]. Values of the root mean square error of approximation (RMSEA) between 0.05 and 0.08 indicate an acceptable fit, below 0.05 indicates an excellent fit [24]. In addition to the internal consistency, the known group validity of the Turkish version of the SPPIC was determined by comparing the mean sum scores for subgroups of participants by using an independent t-test. A significance level of 0.05 was adopted, see below. As there were few missing data items, listwise deletion was adopted in the case of missing values and sum scores were only calculated for those who completed all items of the scale. The following hypotheses were tested:

- Participants who provide family care at least once a week are expected to have a higher self- perceived pressure form informal care as measured by SPPIC than participants who provide family care less than once a week [10].
- Participants who live in the same home as the relative with severe forgetfulness or dementia are expected to have a higher self- perceived pressure form informal care as measured by SPPIC than participants who do not live in the same home as the relative with severe forgetfulness or dementia family [24, 25].
- Participants with no education or only primary education are expected to have a higher self- perceived pressure form informal care as measured by SPPIC than participants who completed secondary or tertiary education [10, 14]. Education is here considered to be an indicator of socioeconomic position.

All analyses were conducted using Stata version 15.0.

## Results

### Pilot test: feasibility, appropriateness and comprehensibility

The content of the questions was considered appropriate by the 30 participants of the pilot test. In addition, the nine questions of the Turkish version of the SPPIC were considered comprehensible by the participants. Furthermore, the length of the questionnaire was evaluated positively and therefore considered feasible for application in research and practice. The pilot test therefore did not reveal any need for further amendments to the Turkish version of the SPPIC.

### Main study: internal consistency and validity

#### Background characteristics

A total of 133 participants with Turkish backgrounds provided family care to loved ones with dementia of whom 117 (89%) completed the Turkish version of SPPIC and could therefore be included in the current analyses. Most of the participants were aged between 36 and 55, were female and had been born in Turkey (see Table 1). A substantial proportion of the participants had no education or had only attended primary school (50.4%). The participants had a greater competence in reading, writing, comprehending and speaking in Turkish than in Dutch (see Appendix 2). Most of the participants (91%) cared for a relative with dementia and few for a friend, neighbour or other person with dementia (9%). More than a third of the participants (38.5%) were living in the same home as the relative with dementia or severe forgetfulness. Domestic help, offering a listening ear and assistance are the most common forms of family care. Around a third of the participants provided family care on a daily basis (see Table 2).

**Table 1:** Sociodemographic sample characteristics (*N* = 117)

*Characteristics*	*Mean (SD)*	*n (%)*
**Sex**		
Female		97 (82.9)
Missing		3 (2.6)
**Age**	45.7 (13.2)	
15–35		23 (19.6)
36–55		65 (55.6)
56–75		22 (18.8)
76–85		2 (1.7)
Missing		5 (4.3)
**Education**^a^		
None or primary school		59 (50.4)
Secondary school		24 (20.5)
SVE^b^		20 (17.1)
HPE or university^c^		9 (7.7)
Other^d^		2 (1.7)
Missing		3 (2.6)
**Brought up in the Netherlands?**		
Yes		15 (12.8)
Missing		2 (1.7)

^a^Education = Highest level of education

^b^SVE = Secondary Vocational Education

^c^HPE or University = Higher professional education or University

^d^Other = Other additional courses

**Table 2:** Features of the relationship between the respondents (*N* = 117) and their relative with dementia

*Characteristics*	*n (%)*
***Dementia in the family?***	
Yes	99 (84.6)
No, but in immediate environment	17 (14.5)
Who is the person with dementia? *	
Partner	22 (18.8)
Child	4 (3.4)
Father (father-in-law)	40 (34.2)
Mother (mother-in-law)	55 (47.0)
Brother or sister	4 (3.5)
Neighbour	13 (11.2)
Different	20 (17.1)
***Do you live together with the person with dementia?***	
Yes	45 (38.5)
Missing	1 (0.9)
***Do you provide help?***	
Yes	117 (100)
Missing	0 (0.0)
***If yes, what kind of help?*** ^a^	
Personal care	25 (21.2)
Domestic help	58 (49.6)
Practical help	51 (43.6)
Listening ear	63 (53.8)
Watching over	41 (35.0)
Nursing care	15 (12.8)
Accompaniment	53 (45.3)
***How often do you provide help?***	
Daily	42 (35.9)
3–6 times a week	15 (12.8)
Up to twice a week	26 (22.2)
< 1 once a week	12 (10.3)
< 1 once a month	15 (12.8)
Missing	7 (5.9)

^a^ *multiple answers possible*

#### Internal consistency of the SPPIC

**Fig. 1:**
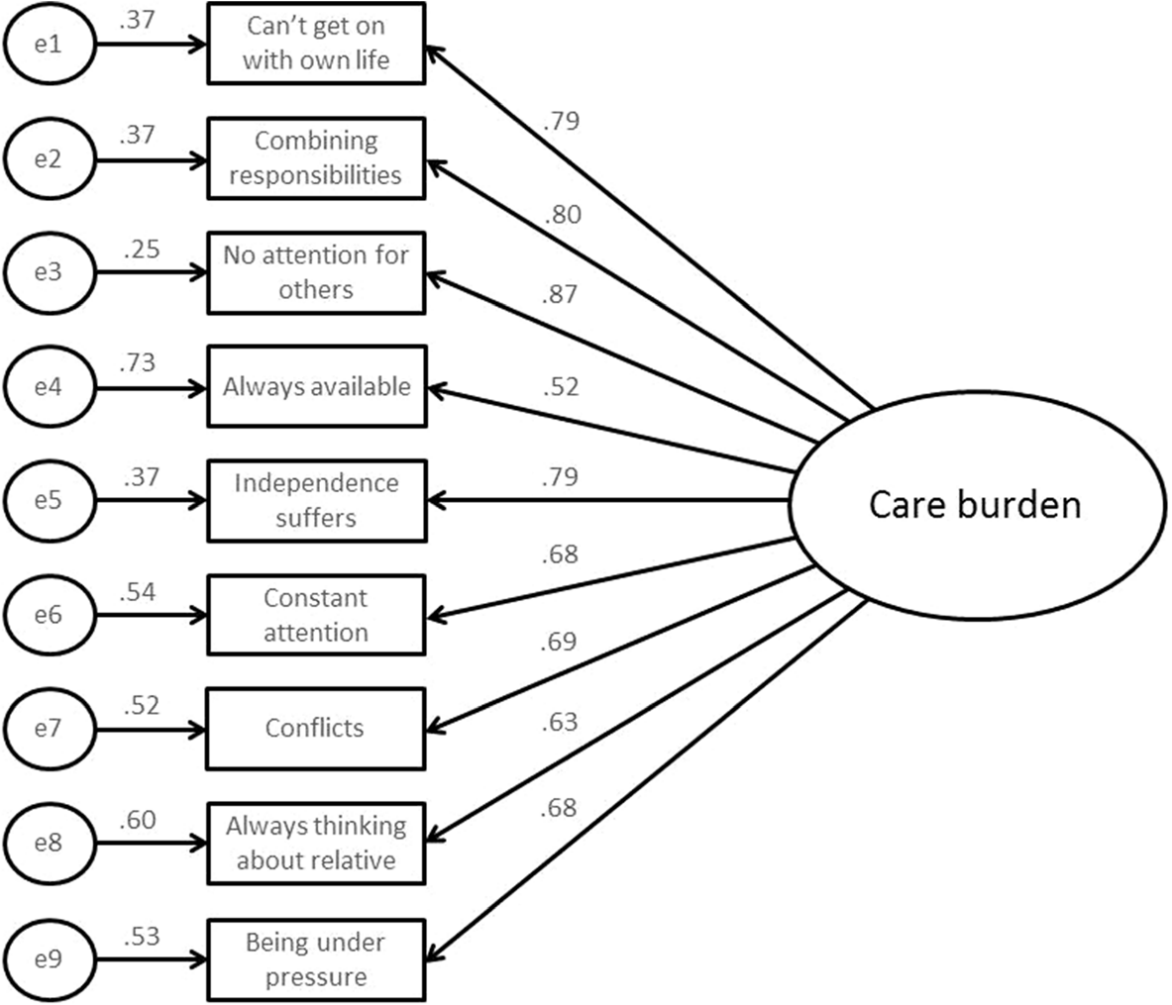
Results of the Confirmatory Factor Analysis for SPPIC including a single factor. χ^2^ = 71.26, *p* = .000; comparative fit index (CFI) = .916; Tucker-Lewis fit index (TLI) = .888; RMSEA = .123

The mean sum score on the SPPIC was 25.8 (SD = 7.9). More detailed information on the scores on the individual items can be found in Appendix 3. The nine items were highly correlated (see Table 3) and showed high internal consistency with a Cronbach’s alpha of 0.94. χ^2^ = 71.26, *p* = .000. The Confirmatory Factor Analysis indicated that the single factor self- perceived pressure form informal care explained varying levels of variance in the items of the SPPIC (ranging from .52 to .87) (see Fig. 1). Most variance was explained in the first three items and the fifth item of the SPPIC. Less variance was explained in the last four items of the SPPIC and the least variance was explained in the fourth item of the SPPIC (see Fig. 1). This implies that factors other than self- perceived pressure form informal care caused variance in the scoring on these items. The comparative fit index (CFI) showed an acceptable model fit (.916), yet the RMSEA (.123) and the Tucker-Lewis fit index (TLI) indicated a lack of fit (.888), as well as the χ^2^ which turned out to be significant (*p* = .000).

**Table 3:** Correlation matrix including the nine items of the Turkish version of the SPPIC

	1	2	3	4	5	6	7	8	9
C1. Owning to the situation of my…I have too little time for myself.	1.00								
C2. Combining the responsibility for my… and for my job and/or family is not easy.	0.70	1.00							
C3. Because of my involvement with my…I don’t pay enough attention to others.	0.71	0.71	1.00						
C4. I must always be available for my…	0.38	0.39	0.40	1.00					
C5. My independence is suffering	0.64	0.64	0.69	0.44	1.00				
C6. The situation of my … constantly demands my attention	0.49	0.50	0.55	0.58	0.59	1.00			
C7. Because of my involvement with my…I am getting into conflict at home or at work.	0.49	0.52	0.60	0.36	0.59	0.43	1.00		
C8. The situation of my…is a constant preoccupation	0.46	0.45	0.56	0.37	0.36	0.54	0.51	1.00	
C9. Generally speaking I feel very pressured by the situation of my…	0.52	0.49	0.58	0.30	0.51	0.45	0.54	0.64	1.00

#### Known group validity

In line with the expectations, there was an association between the frequency of caregiving and self- perceived pressure form informal care: family caregivers who provided care at least once a week to a relative with severe forgetfulness or dementia perceived a greater pressure from informal care (M = 26.6, SD = 7.7) than those who offered care less than once a week (M = 21.9, SD = 7.1; t (100) = 2.76, *p* = 0.007). Also in line with the expectations, family caregivers who shared a home with the relative with severe forgetfulness or dementia experienced a greater pressure form informal care (M = 28.9, SD = 7.3) than those who did not (M = 23.8, SD = 7.7; t (105) = 3.37, *p* = 0.001). However, contrary to what we expected, people who had completed no education or had only been through primary school did not have a higher self- perceived pressure form informal care (M = 26.5, SD = 7.3) than those who completed secondary or tertiary education (M = 25.1, SD = 8.6; t (103) = 0.89, *p* = 0.378).

## Discussion

The aim of the current study was to evaluate the internal consistency and validity of the Turkish translation of the SPPIC. The SPPIC is a measurement instrument, originally developed and validated in Dutch, to assess the self- perceived pressure form informal care among family caregivers [26]. A pilot test was conducted to obtain insights into the feasibility, comprehensibility and appropriateness of the translated items of the Turkish SPPIC. All participants of the pilot test found the translated items of the SPPIC comprehensible, appropriate and feasible.

After the pilot test, a validation study was conducted to evaluate the internal consistency and validity of the Turkish translation of the SPPIC. The number of missing answers was low, which indicates that the participants understood the questions and were motivated to fill in the whole questionnaire. Research among ethnic minority populations is characterised by relatively high attrition rates [27]. In order to prevent both attrition and missing values, we applied various strategies: involving people with the same cultural background in the design of the research, pre-testing the questions and explaining in detail how the questionnaire should be completed.

Where study participants had the option of choosing between the Dutch and the Turkish versions of the SPPIC, a vast majority of participants chose the Turkish version, even though most of the participants were aged 55 or younger, and had often lived most of their life in the Netherlands. This finding is all the more relevant in the context of offering educational interventions to people with a Turkish immigration background. It is sometimes assumed that the second generation have a good command of Dutch but that when, as in this study, a choice is offered between completing a written questionnaire in Turkish or Dutch, the majority of the participants opt for the Turkish questionnaire. It is therefore recommended that the language preferences of the target group should be taken into account.

The internal consistency of the nine items of the SPPIC could be considered good based on the Cronbach’s alpha. However, the outcomes of the Confirmatory Factor Analysis, testing a single factor solution, indicated an overall moderate model fit, which could imply that a multiple factor solution might better fit the data. Although all items seemed to measure an aspect of self-perceived pressure from informal care, not all variance in the item scores could be explained by the underlying factor ‘self-perceived pressure from informal care’. This especially applied for the item “I must always be available for my […]”, which suggests that factors other than ‘self-perceived pressure from informal care’ might better explain variation in the scoring on these items. The strongest indicators of ‘self-perceived pressure from informal care’ seem to be the items that assess perceptions with respect to getting on with life (item 1); combining responsibilities (item 2); giving enough attention to others (item 3); personal independence (item 5). Most variance in these items can be explained by ‘self-perceived pressure from informal care’.

A possible explanation for the moderate fit of the single factor solution, is that self-perceived pressure from informal care aspects as addressed in the nine items of the EDIZ, are better indicators of self-perceived pressure from informal care in family caregivers with a Dutch background than in family caregivers with a Turkish migration background. When comparing the outcomes of our validation study with the outcomes of the validation study of the original (Dutch) version of the EDIZ, there are some notable differences in how participants responded to the nine items. Pot and colleagues [17] listed the nine items, with at top of the list the item that most participants agreed with (and that are therefore assumed to require the least pressure in order to make them agree) and at the bottom of the list the item that fewest participants agreed with (and therefore required the most pressure in order to make them agree). When listing the items based on the outcomes of our study according to the proportion that agreed with an item, we see a slightly different order (see Appendix 4). The main notable difference between our list and the list as presented by Pot and colleagues [17], is that relatively many participants in their study agreed with the item “Owning to the situation of my….I have too little time for myself”, whereas in our study we found that few people agreed with this item. This suggests that family caregivers with a Dutch background feel that their care duties start interfering with their life at an earlier stage than caregivers with a Turkish background.

In addition, our findings imply that agreement with the item “I must always be available for my […]” cannot be explained well by the latent variable ‘self-perceived pressure from informal care’. It could be that family caregivers with Turkish background might strongly agree with the statement that they always have to be available for their relative with dementia, regardless of the self-perceived pressure from informal care. This assumption is supported by the finding that the largest proportion of participants agreed with this item, perhaps including those who perceived little pressure. Among caregivers with a Dutch background, agreeing with this statement might be more strongly associated with a higher self-perceived pressure from informal care.

Based on these findings, more research is recommended on aspects that should be measured in order to obtain a more comprehensive insight into self-perceived pressure from informal care in family caregivers with a Turkish immigrant background.

In line with other studies [1–3, 11, 28–30], the current study showed that the intensity of providing family care is associated with the self-perceived pressure from informal care: frequently providing care is associated with a higher self-perceived pressure from informal care and this is even more so for spouses of a person with dementia. This is a relevant finding because providing family care is seen in Turkish immigrant communities as a task provided primarily by women [20].To prevent psychological and physical health problems in family caregivers [1–3, 11–13], it is important to signal a high self-perceived pressure from informal care in family caregivers.

Little is known about the self-perceived pressure from informal care and possible health effects in ethnic minorities. The SPPIC could be used to obtain more insights in this respect. However, a limitation of this study is that it only focuses on the validation of a Turkish translation of the SPPIC. For future research regarding family caregiving in ethnic minorities, it is recommended that there should be an evaluation of whether the SPPIC should be translated and validated in the mother tongues of other ethnic minority groups. As some languages are largely phonetic (Moroccan Berber), the main language of the country of residence might be more applicable for some ethnic minority groups.

Another limitation of this study is that the majority of the participants were female and it was not known if family caregivers were assisted in caring for the person with dementia by healthcare professionals (for instance home care) or other family caregivers. More research is recommended into the validation of the (Turkish) SPPIC among larger groups of male caregivers and to get a better understanding of the level of professional or family support received.

Furthermore we would recommend that additional studies be carried out using larger samples of participants in order to further document the validity and responsiveness of the Turkish SPPIC. Finally, it is also important to test Turkish version of SPPIC in other western European countries that are home to large communities of Turkish migrants (for example Flanders in Belgium, and France and Germany). Turkish migrants living in these countries have similar background characteristics, migration history and socioeconomic conditions to the migrants in the present study.

### Conclusion

The Turkish translation of the SPPIC can be considered a feasible and valid measurement instrument to assess self-perceived pressure from informal care among family caregivers with a Turkish immigrant background, caring for a person with dementia living in the Netherlands. Four out of the nine items of the SPPIC seem specifically to be strong indicators of self-perceived pressure from informal care. The Turkish translation of the SPPIC can be used in future research and practice, to obtain insight into the (more intensive) support needs in the care for a loved one with dementia. At the same time it is recommended to conduct more research on how the measurement of self-perceived pressure from informal care among family caregivers with a Turkish immigrant background can be further improved.

## Supplementary Information

**Additional file 1: Appendix 1.** SPPIC questionnaire (Dutch and English). **Appendix 2.** Con characteristics relating to language skills. **Appendix 3.** Missing values, mean, skewness and kurtosis for the Turkish translation of the SPPIC per item. **Appendix 4.** Items in order of proportion that agreed with the items.

## Data Availability

The datasets used and/or analysed during the current study available from the corresponding author on reasonable request.

## Abbreviations

SPPIC: Self-perceived Pressure from Informal Care
CFA: Confirmatory factor analysis
CFI: Comparative Fit Index
TLI: Tucker-Lewis index
RMSEA: Values of the root mean square error of approximation
