# Supplementary material for: The Turkish version of the SPPIC validated among informal caregivers with a Turkish immigrant background
Source: BMC Geriatr. 2021 Apr 29;21:284. doi: 10.1186/s12877-021-02161-6 (PMC8086099; doi:10.1186/s12877-021-02161-6)
Supplement: Supplementary file 1 — Additional file 1: Appendix 1. SPPIC questionnaire (Dutch and English). Appendix 2. Con characteristics relating to language skills. Appendix 3. Missing values, mean, skewness and kurtosis for the Turkish translation of the SPPIC per item. Appendix 4. Items in order of proportion that agreed with the items. [file 12877_2021_2161_MOESM1_ESM.zip › Appendix 1-3 BMC_EDIZR5.docx]

**Appendix 1. Missing values, mean, skewness and kurtosis for the Turkish translation of the EDIZ per item**

| Items of the EDIZ | Scoring on the items | | | | | | |
| --- | --- | --- | --- | --- | --- | --- | --- |
|  | Missing | Mean | SD | Min | Max | Skewness | Kurtosis |
| C1. Owning to the situation of my….I have too little time for myself. | 3 | 2.52 | 1.11 | 1 | 5 | 0.329 | 2.37 |
| C2. Combining the responsibility for my…. and for my job and/or family is not easy. | 4 | 2.79 | 1.23 | 1 | 5 | 0.120 | 1.92 |
| C3. Because of my involvement with my….I don’t pay enough attention to others. | 2 | 2.57 | 1.16 | 1 | 5 | 0.299 | 2.19 |
| C4. I must always be available for my….. | 4 | 3.50 | 1.20 | 1 | 5 | -0.777 | 2.69 |
| C5. My independence is suffering | 3 | 2.82 | 1.21 | 1 | 5 | 0.041 | 1.98 |
| C6. The situation of my …. constantly demands my attention | 3 | 3.36 | 1.20 | 1 | 5 | -0.534 | 2.42 |
| C7. Because of my involvement with my….I am getting into conflict at home or at work. | 3 | 2.42 | 1.14 | 1 | 5 | 0.542 | 2.39 |
| C8. The situation of my…..is a constant preoccupation | 3 | 3.17 | 1.11 | 1 | 5 | -0.371 | 2.67 |
| C9. Generally speaking I feel very pressured by the situation of my….. | 2 | 2.74 | 1.12 | 1 | 5 | 0.188 | -0.715 |

**Appendix 2. EDIZ questionnaire (Dutch and English)**

**
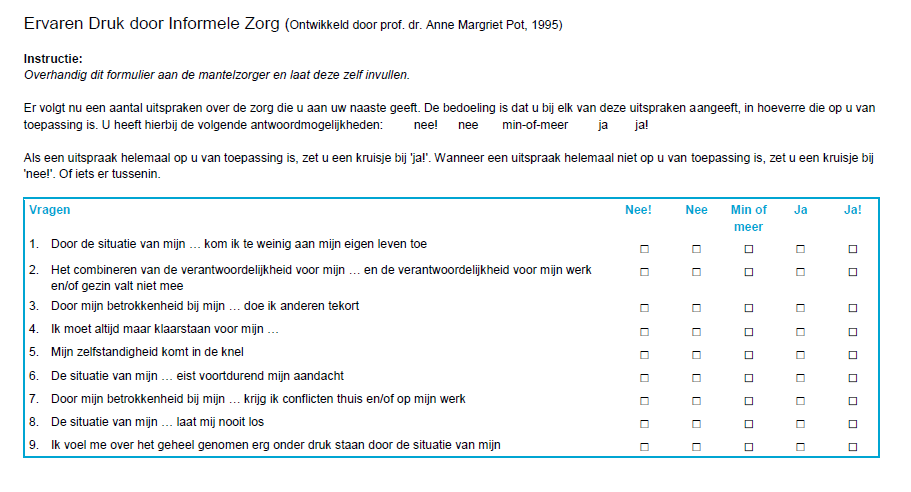
**

Perceived Burden of Family Care (Developed by Prof. Anne Margriet Pot. 1995)

**Instructions:**

*Hand this form to the family-based carer and get them to fill it in themselves.*

You will see a number of statements below about the care that you provide for your relative. The idea is that should indicate how much each of these statements applies in your case. You have the following possible answers for this:

no!

no

more or less

yes

yes!

If a statement applies very much to you, put a cross in the “yes!” column. If a statement does not apply to you at all, put a cross in the “no!” column, or somewhere in between.

|  | No! | no | more or less | yes | yes! |
| --- | --- | --- | --- | --- | --- |
| C1. Owning to the situation of my….I have too little time for myself. |  |  |  |  |  |
| C2. Combining the responsibility for my…. and for my job and/or family is not easy. |  |  |  |  |  |
| C3. Because of my involvement with my….I don’t pay enough attention to others. |  |  |  |  |  |
| C4. I must always be available for my….. |  |  |  |  |  |
| C5. My independence is suffering |  |  |  |  |  |
| C6. The situation of my …. constantly demands my attention |  |  |  |  |  |
| C7. Because of my involvement with my….I am getting into conflict at home or at work. |  |  |  |  |  |
| C8. The situation of my…..is a constant preoccupation |  |  |  |  |  |
| C9. Generally speaking I feel very pressured by the situation of my….. |  |  |  |  |  |

**Appendix 3.**

**con characteristics relating to language skills**

|  | **Turkish (N=117)** | | | |
| --- | --- | --- | --- | --- |
|  | None | Little | Good | Missing |
| DU Understanding | 4 (3.4) | 73 (57.4) | 39 (33.3) | 1 (0.9) |
| DU Speaking | 5 (4.3) | 71 (60.7) | 39 (33.3) | 2 (1.7) |
| DU Reading | 14 (12.0) | 60 (51.2) | 43 (36.8) | 0 (0) |
| DU Writing | 23 (19.7) | 55 (47.0) | 39 (33.3) | 0 (0) |
|  |  |  |  |  |
| MT Understanding | 3 (2.6) | 29 (24.7) | 81 (69.2) | 4 (3.4) |
| MT Speaking | 2 (1.7) | 33 (28.2) | 81 (69.2) | 1 (0.9) |
| MT Reading | 7 (6.0) | 32 (27.4) | 78 (66.7) | 0 (0) |
| MT Writing | 9 (7.7) | 37 (31.6) | 71 (60.7) | 0 (0) |

DU=Dutch; MT=mother tongue
