# Supplementary material for: The Turkish version of the SPPIC validated among informal caregivers with a Turkish immigrant background
Source: BMC Geriatr. 2021 Apr 29;21:284. doi: 10.1186/s12877-021-02161-6 (PMC8086099; doi:10.1186/s12877-021-02161-6)
Supplement: Supplementary file 1 — Additional file 1: Appendix 1. SPPIC questionnaire (Dutch and English). Appendix 2. Con characteristics relating to language skills. Appendix 3. Missing values, mean, skewness and kurtosis for the Turkish translation of the SPPIC per item. Appendix 4. Items in order of proportion that agreed with the items. [file 12877_2021_2161_MOESM1_ESM.zip › Appendix 4R5.docx]

**Appendix 4. Items of the EDIZ in order of proportion that agreed**

| **Items of the EDIZ in order of proportion that agreed with the items ranging from the highest to the lowest proportion** | | |
| --- | --- | --- |
| *Item* | *Based on the outcomes of the validation study of the Dutch EDIZ (see Pot e.a., 1998^a^)* | *Based on the outcomes of this validation study of the Turkish version of the EDIZ* |
| 1 | The situation of my … constantly demands my attention | I must always be available for my… |
| 2 | The situation of my…is a constant preoccupation | The situation of my … constantly demands my attention |
| 3 | I must always be available for my… | The situation of my…is a constant preoccupation |
| 4 | Owning to the situation of my…I have too little time for myself. | Combining the responsibility for my… and for my job and/or family is not easy. |
| 5 | Generally speaking I feel very pressured by the situation of my… | My independence is suffering |
| 6 | Combining the responsibility for my… and for my job and/or family is not easy. | Generally speaking I feel very pressured by the situation of my… |
| 7 | My independence is suffering | Because of my involvement with my…I don’t pay enough attention to others. |
| 8 | Because of my involvement with my…I don’t pay enough attention to others. | Because of my involvement with my…I am getting into conflict at home or at work. |
| 9 | Because of my involvement with my…I am getting into conflict at home or at work. | Owning to the situation of my…I have too little time for myself. |

^a^ Pot, A.M., van Dyck, R., Deeg, D.J.H. (1995). Ervaren druk door informele zorg; constructie van een schaal. Tijdschrift voor Gerontologie en Geriatrie 26, p 214 – 219.
